# Supplementary material for: Human interleukin-4-dependent facilitation of human IgG production in PBL-NOG-hIL-4-Tg mice
Source: Front Immunol. 2025 Nov 26;16:1670682. doi: 10.3389/fimmu.2025.1670682 (PMC12690289; doi:10.3389/fimmu.2025.1670682)
Supplement: Supplementary file 1 [file DataSheet1.pdf]

Human Interleukin-4-dependent facilitation of anti-peptide Ig production with antibody class switching and diverse B and T cell repertoires

Yoshie Kametani[1,2*], Shino Ohshima[1], Ryoji Ito[3], Yusuke Ohno[3], Soga Yamada[1], Yuki Hoshino[1], Asuka Miyamoto[1], Mao Suzuki[1], Nagi Katano[1] Banri Tsuda[4], Mariko Miyazawa[5] , Hirofumi Kashiwagi[5], Daiki Kirigaya[1], Tomoka Shimizu[1], Mika Kojima[1], Yusuke Kikuchi[1], Shunsuke Nakada[1], Rentaro Ohki[1], Atsushi Yasuda[6], Ayako Hirota[7], Toshiro Seki[6], Yukio Nakamura[8] , Jerzy K. Kulski[1,9] , Tomotaka Mabuchi[7], Hitoshi Ishimoto[5], and Takashi Shiina[1,2]

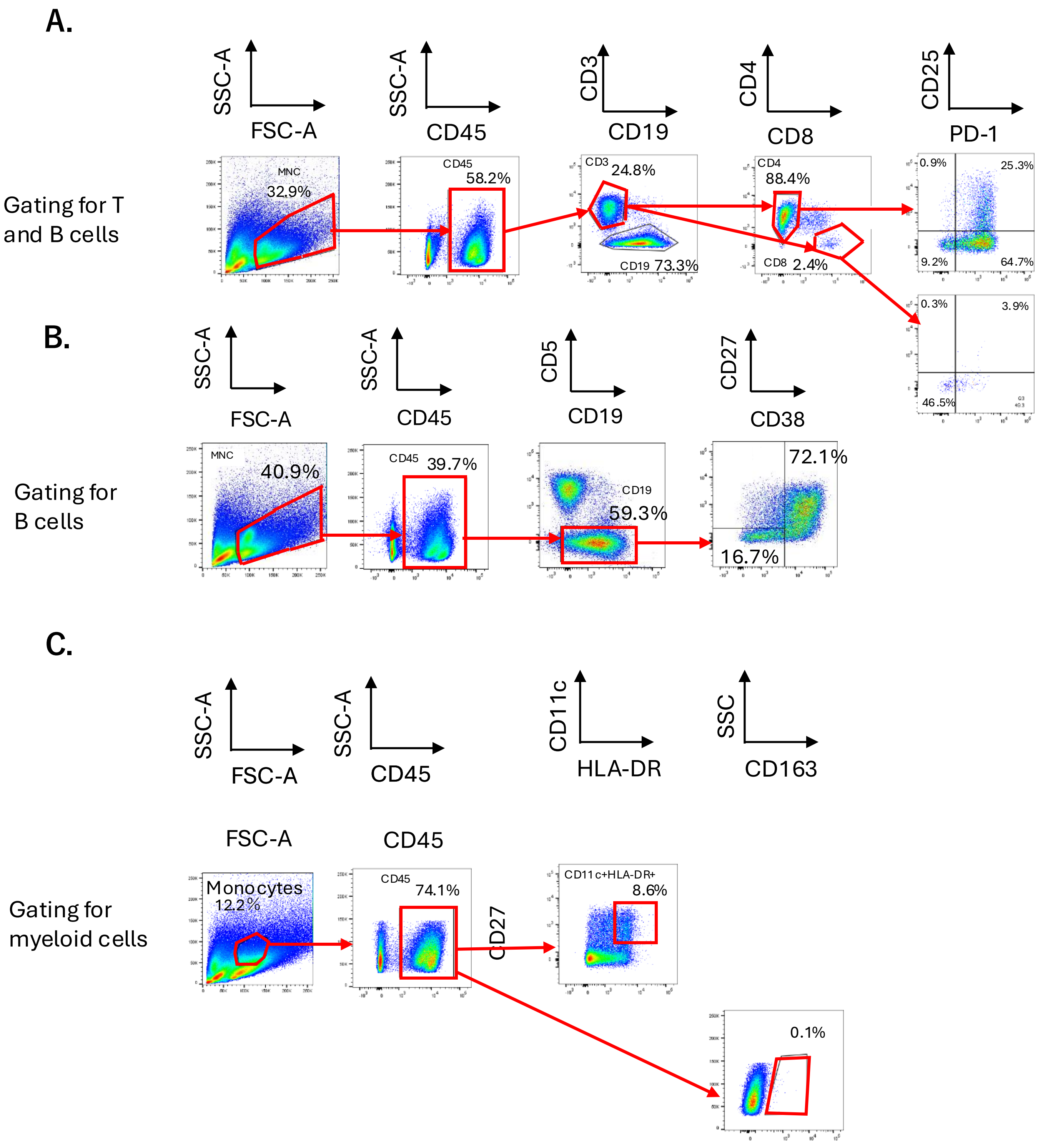

Supplemental Figure 1 Lymphocyte gating

(A) Gating strategy for T cell subsets and B cells with activation markers in PBL-NOG-hIL-4-Tg spleen cells. Cells were gated within the mononuclear cell gate (FSC: 80K–250K). (B) Gating strategy for distinguishing plasmablasts among B cells in PBL-NOG-hIL-4-Tg spleen cells. Cells were gated within the mononuclear cell gate (FSC: 80K–250K). Red squares: Gated cell populations. Red arrows: Indicate the next gating steps. (C) Gating strategy for dendritic cells (DCs) and macrophages in PBL-NOG-hIL-4-Tg spleen cells. Red squares: Gated cell populations. Red arrows: Indicate the next gating steps.

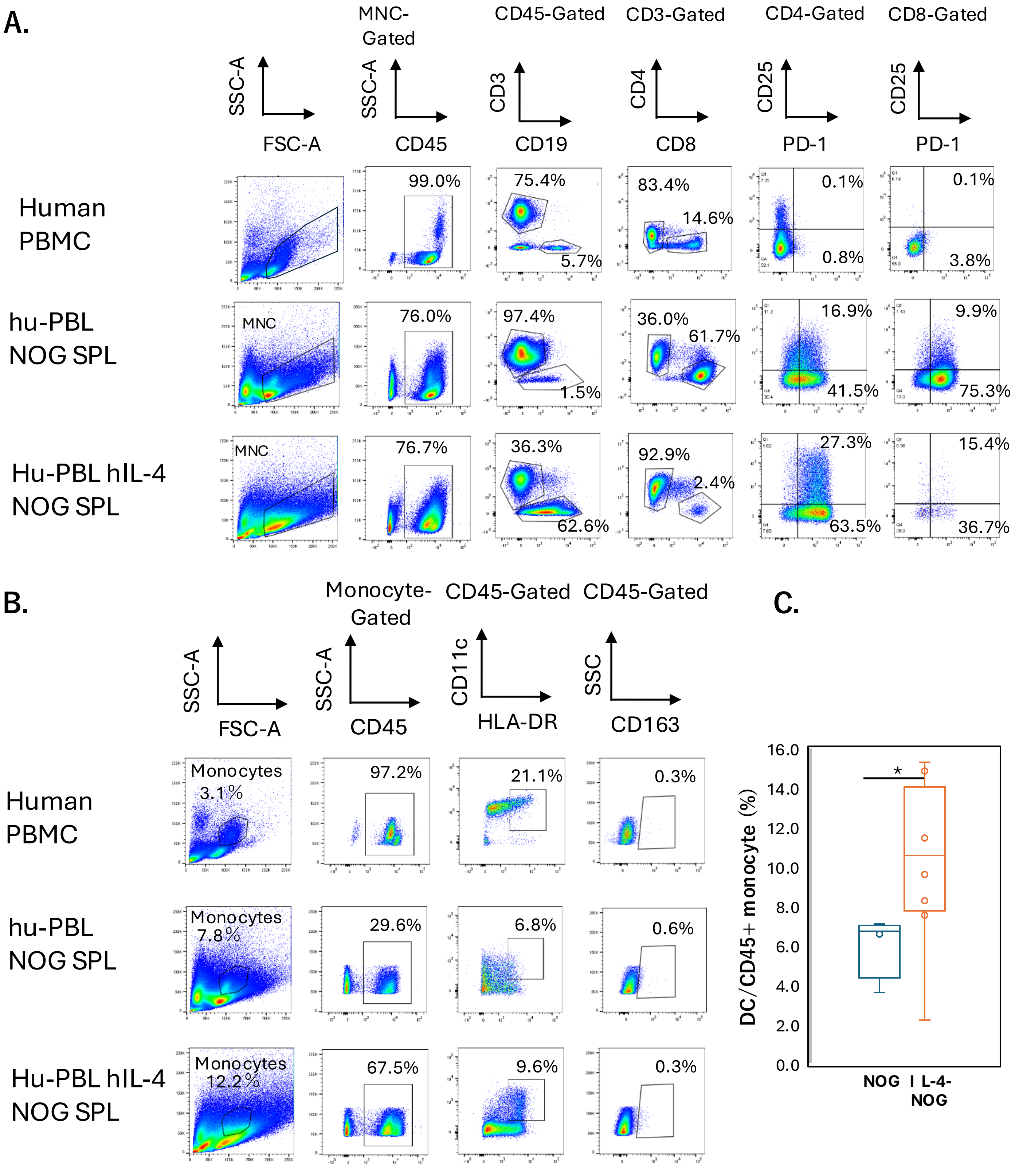

Supplemental Figure 2 Flow cytometry of engrafted T Cells and myeloid cells in hu-PBL hIL-4 NOG mice.

(A) Representative flow cytometry (FCM) patterns of lymphocyte-gated PBMCs and mouse spleen cells. Cells were further gated for $CD45^+$ populations, T cell markers, and activation/exhaustion markers.

(B) Monocyte-gated PBMCs and mouse spleen cells were analyzed for $CD45^+$ populations. DCs were identified as $CD11c^+HLA\text{-}DR^+$ cells, and macrophages as $CD163^+$ cells. Upper panels: Original PBMCs. Middle panels: Spleen cells from NOG-engrafted mice. Lower panels: Spleen cells from NOG-hIL-4-Tg-engrafted mice. The percentages of gated subsets are shown in each panel. (C) Mean percentage of DCs in NOG versus NOG-hIL-4-Tg spleens (IL-4-NOG). NOG ($n = 4$), NOG-hIL-4-Tg ($n = 8$).Student's *t*-test was performed, with significance indicated as follows: $P < 0.05$ (*).

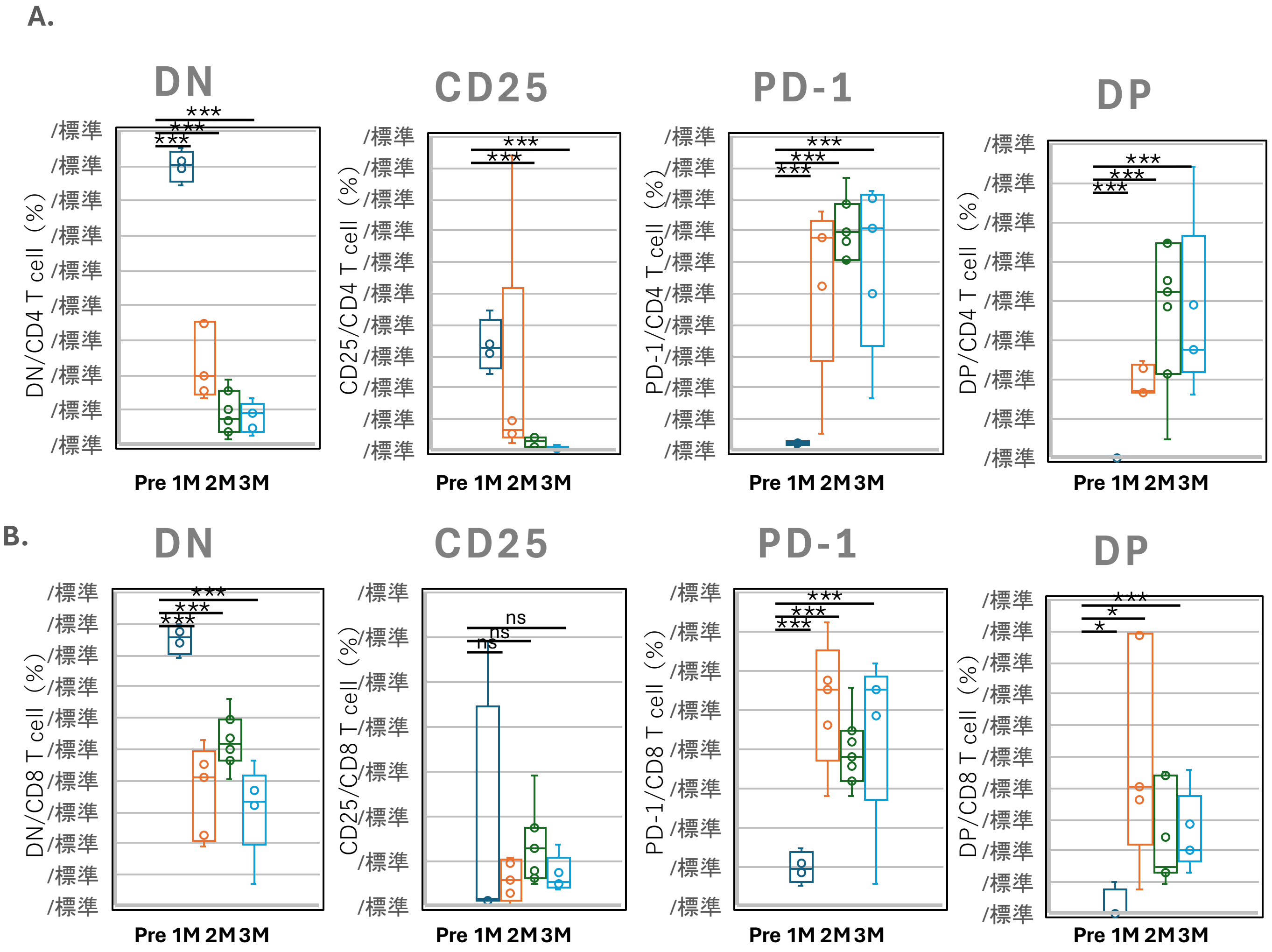

Supplemental Figure 3. CD25 and PD-1 expression on engrafted human T cells in NOG-hIL-4-Tg mice.

(A) $CD4^{+}$ T cells, (B) $CD8^{+}$ T cells. Double-negative (DN): $CD25^{-}PD\text{-}1^{-}$ double-negative T cells, CD25: $CD25^{+}$ single-positive T cells, PD-1: $PD\text{-}1^{+}$ single-positive T cells, Double-positive (DP): $CD25^{+}PD\text{-}1^{+}$ double-positive T cells. Time points: Pre (n = 4), 1M (n = 7), 2M (n = 7), 3M (n = 3). TIK107 and TIK113 were not analyzed due to insufficient cell numbers for flow cytometry. Data are presented as mean ± S.D. Student's *t*-test was performed, with significance indicated as follows: $P < 0.05$ (*), $P < 0.001$ (**), ns = not significant. Sample details are provided in Table 1B.

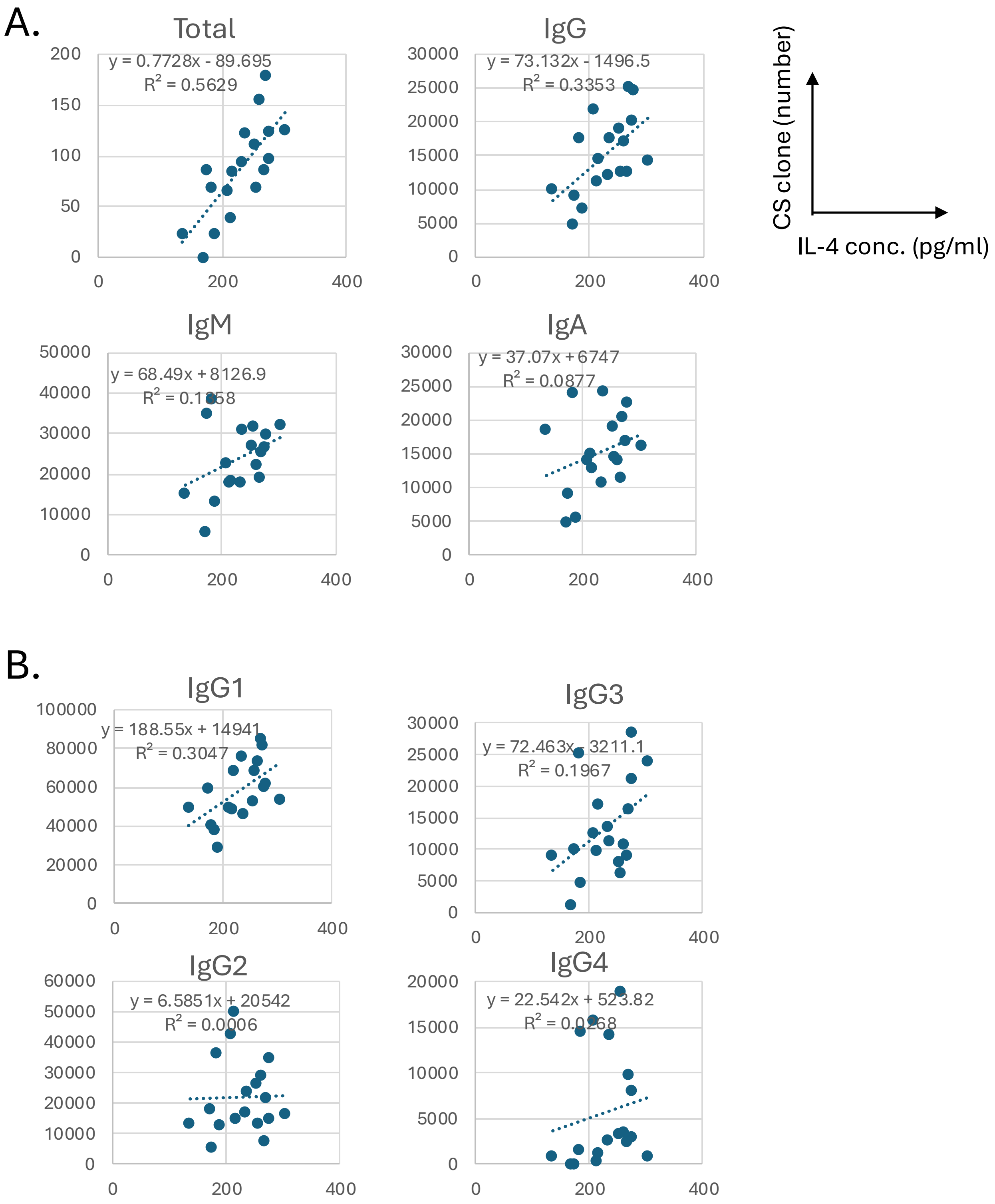

Supplemental Figure 4. Correlation between IL-4 concentration and the number of class-switched clones

(A) Total clones (>0.01% of total), IgM, IgG, IgA.
(n = 18). Samples with low IL-4 levels (<100 pg/mL; TIK110 and TIK112) and high IL-4 levels (>500 pg/mL; TIK105) were excluded. The regression lines, formulas, and R^2 values are displayed in each panel. F-values for each marker: Total clones = 0.0003349, IgM = 0.1323, IgG = 0.0118, IgA = 0.2329.
(B) Total clones (>0.01% of total), (B) IgM, (C) IgG, (D) IgA.
(n = 18). Samples with low IL-4 levels (<100 pg/mL; TIK110 and TIK112) and high IL-4 levels (>500 pg/mL; TIK105) were excluded. The regression lines, formulas, and R^2 values are displayed in each panel. F-values for each marker: IgG1= 0.017, IgG2 =0.9229, IgG3 = 0.0653Ig4 = 0.5159.

A.

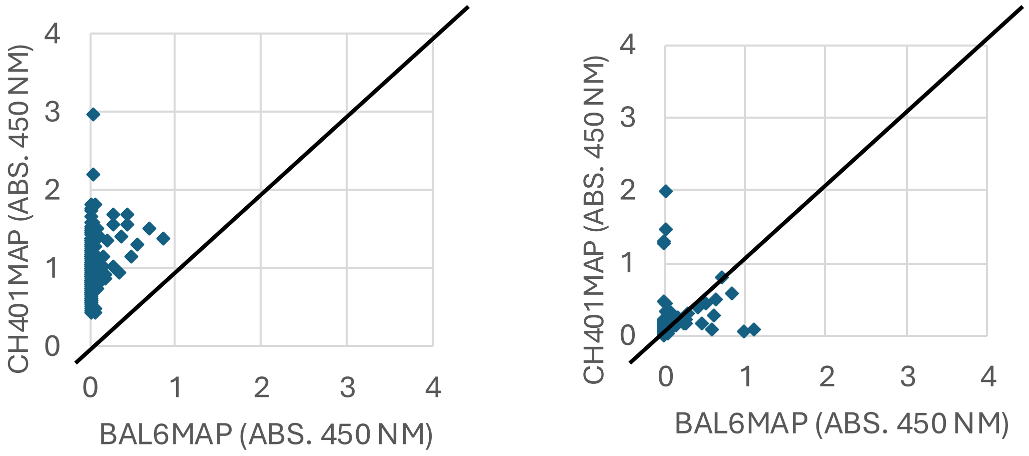

B.

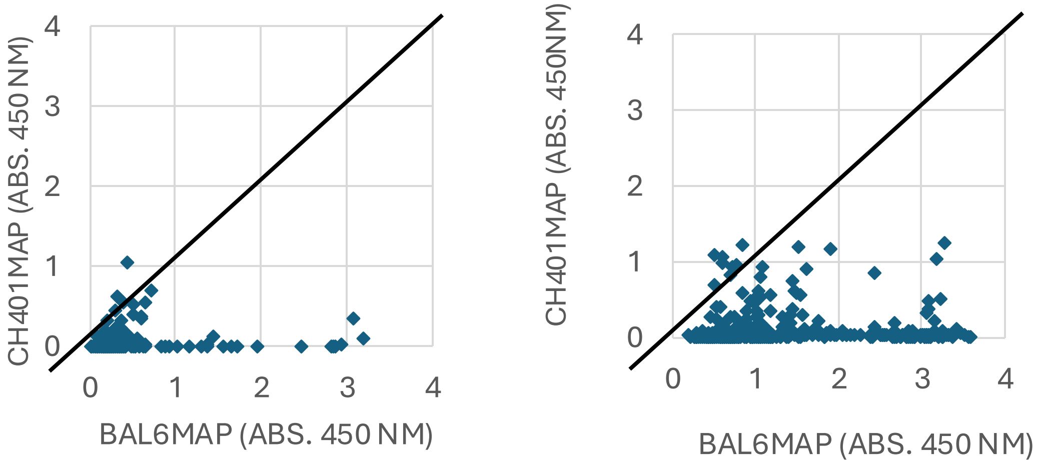

Supplemental Figure 5. Antigen-specific clones produced by BALB/c mice

(A) CH401MAP-immunized mouse clones (n = 2). Left panel: BALB1, Right panel: BALB2. The antibody titer for both mice was greater than 1:1,000.
(B) BAL6MAP-immunized mouse clones (n = 2). Left panel: BALB3, Right panel: BALB4. The antibody titer for both mice was greater than 1:100,000. Each dot represents a single clone.
