# Supplementary material for: Human interleukin-4-dependent facilitation of human IgG production in PBL-NOG-hIL-4-Tg mice
Source: Front Immunol. 2025 Nov 26;16:1670682. doi: 10.3389/fimmu.2025.1670682 (PMC12690289; doi:10.3389/fimmu.2025.1670682)
Supplement: Supplementary file 3 [file Presentation1.pptx]

## Slide 1
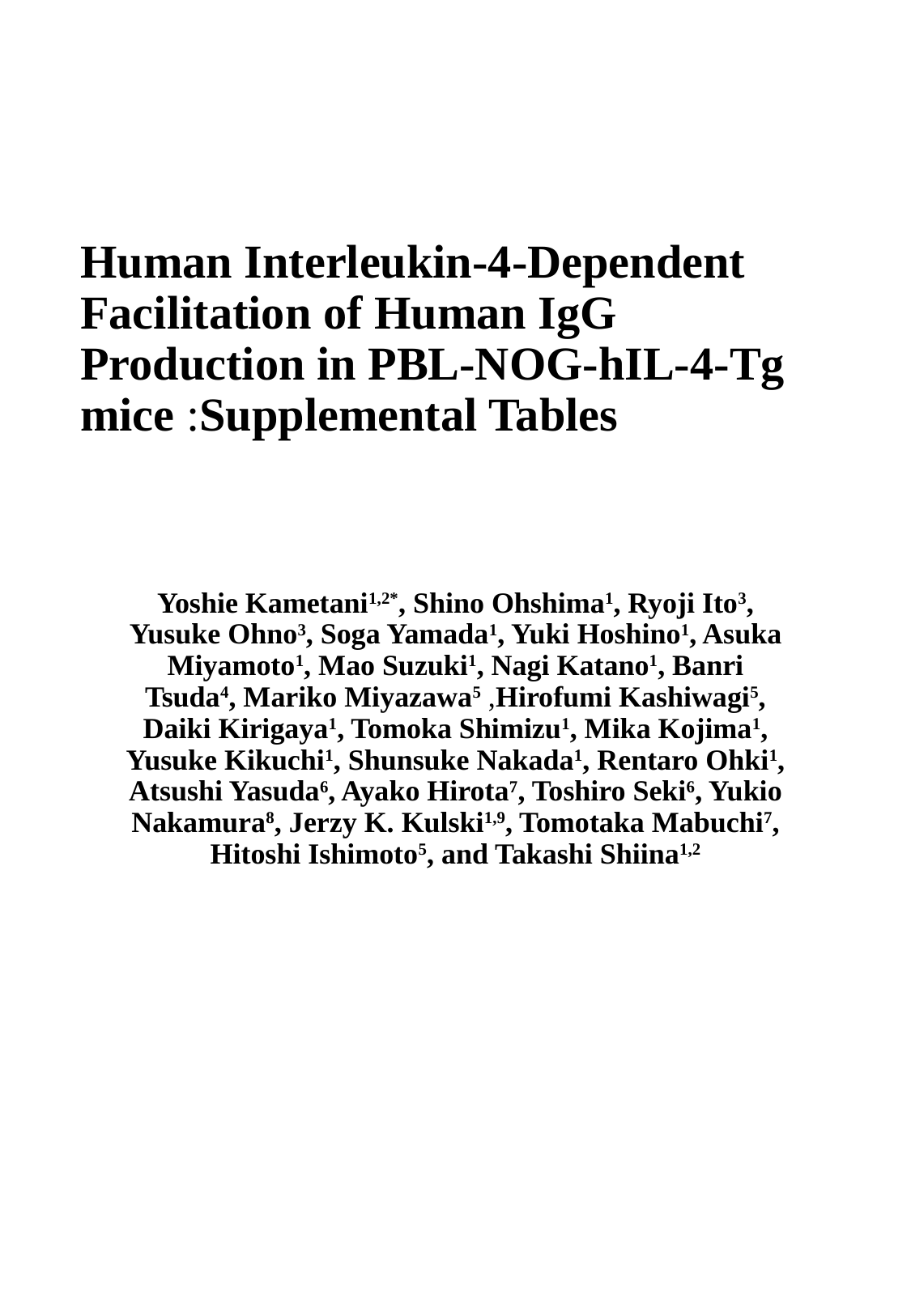

# Human Interleukin-4-Dependent Facilitation of Human IgG Production in PBL-NOG-hIL-4-Tg mice :Supplemental Tables
Yoshie Kametani1,2*, Shino Ohshima1, Ryoji Ito3, Yusuke Ohno3, Soga Yamada1, Yuki Hoshino1, Asuka Miyamoto1, Mao Suzuki1, Nagi Katano1, Banri Tsuda4, Mariko Miyazawa5 ,Hirofumi Kashiwagi5, Daiki Kirigaya1, Tomoka Shimizu1, Mika Kojima1, Yusuke Kikuchi1, Shunsuke Nakada1, Rentaro Ohki1, Atsushi Yasuda6, Ayako Hirota7, Toshiro Seki6, Yukio Nakamura8, Jerzy K. Kulski1,9, Tomotaka Mabuchi7, Hitoshi Ishimoto5, and Takashi Shiina1,2

## Slide 2
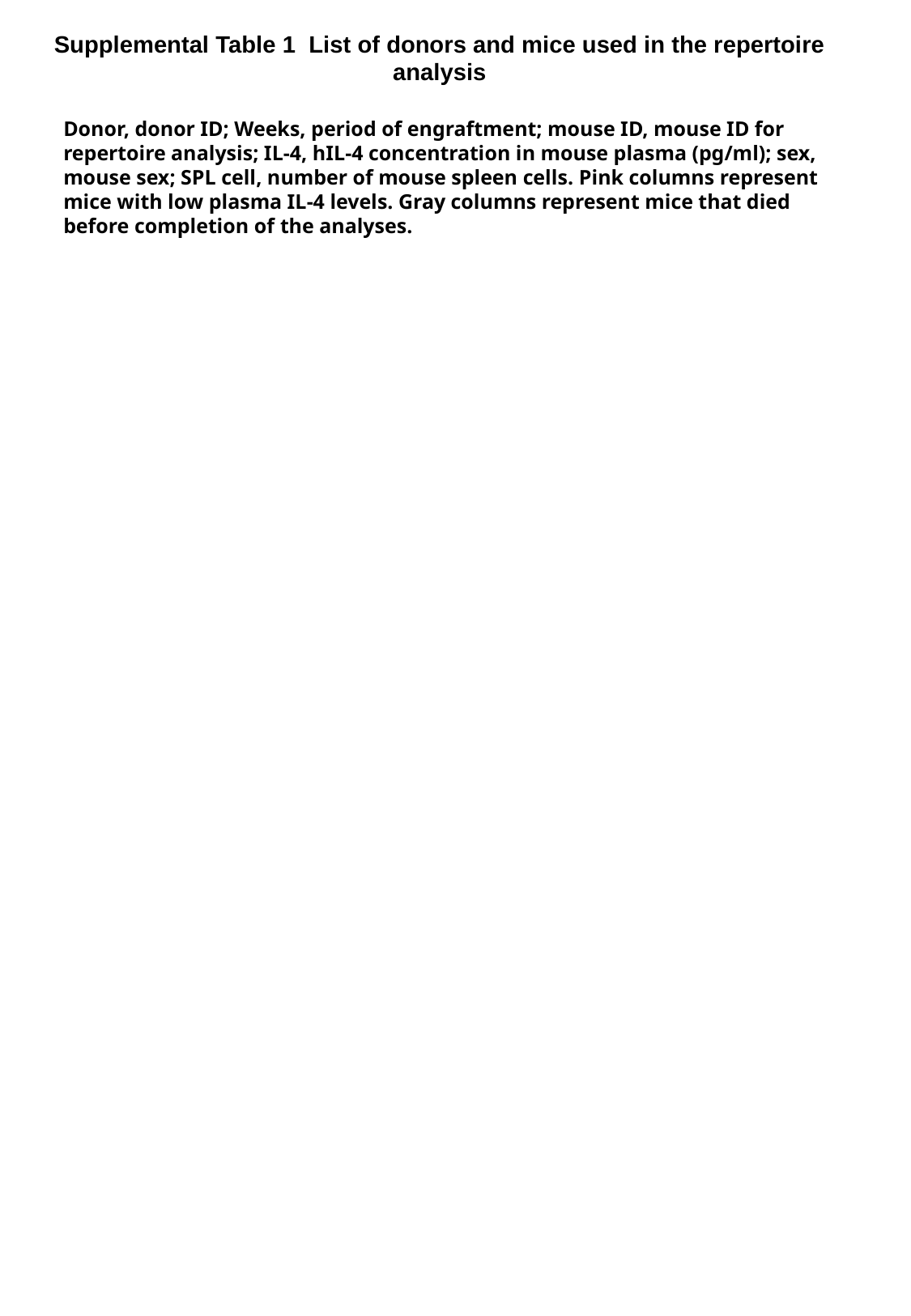

Supplemental Table 1 List of donors and mice used in the repertoire analysis
Donor, donor ID; Weeks, period of engraftment; mouse ID, mouse ID for repertoire analysis; IL-4, hIL-4 concentration in mouse plasma (pg/ml); sex, mouse sex; SPL cell, number of mouse spleen cells. Pink columns represent mice with low plasma IL-4 levels. Gray columns represent mice that died before completion of the analyses.

## Slide 3
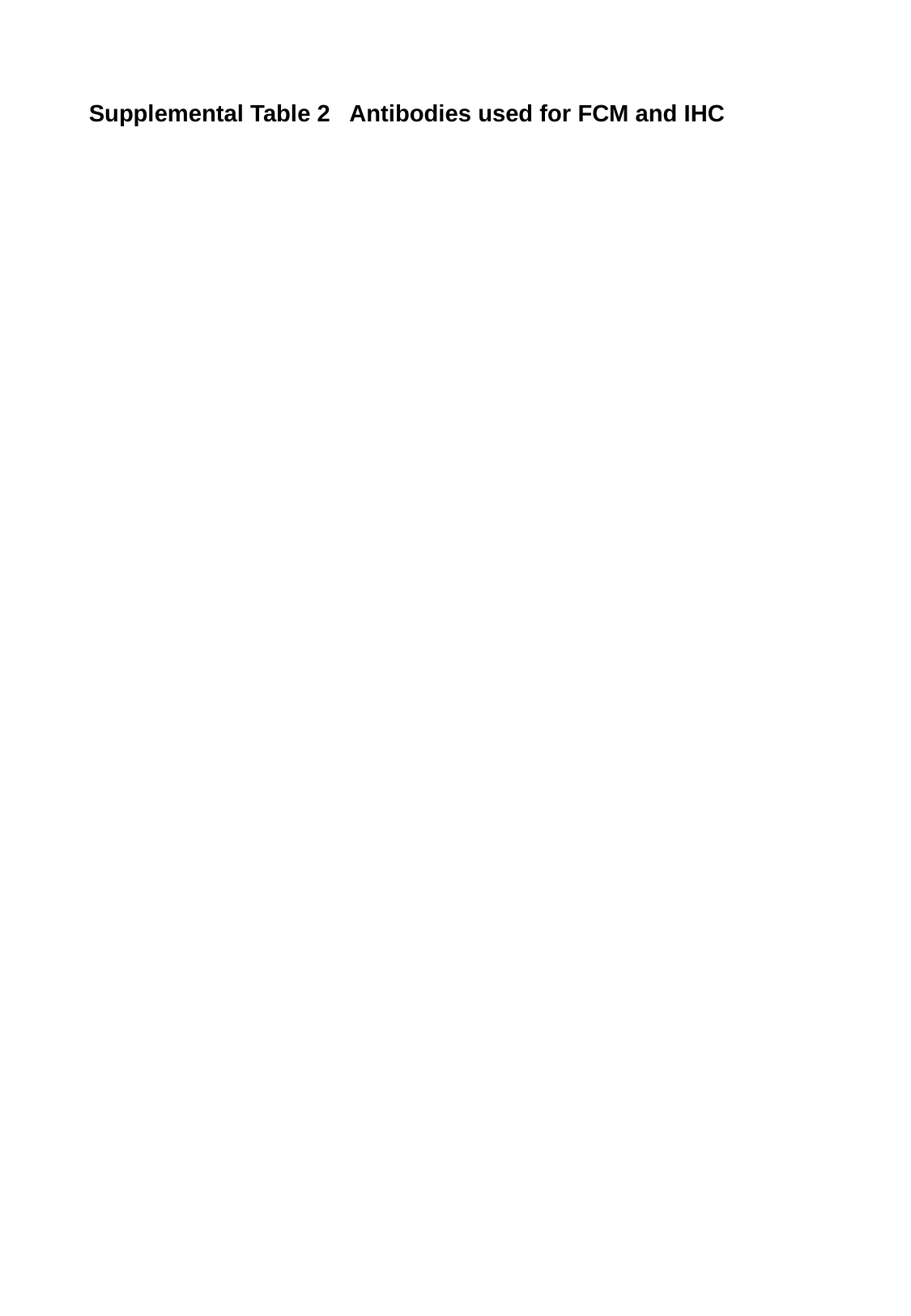

Supplemental Table 2 Antibodies used for FCM and IHC

## Slide 4
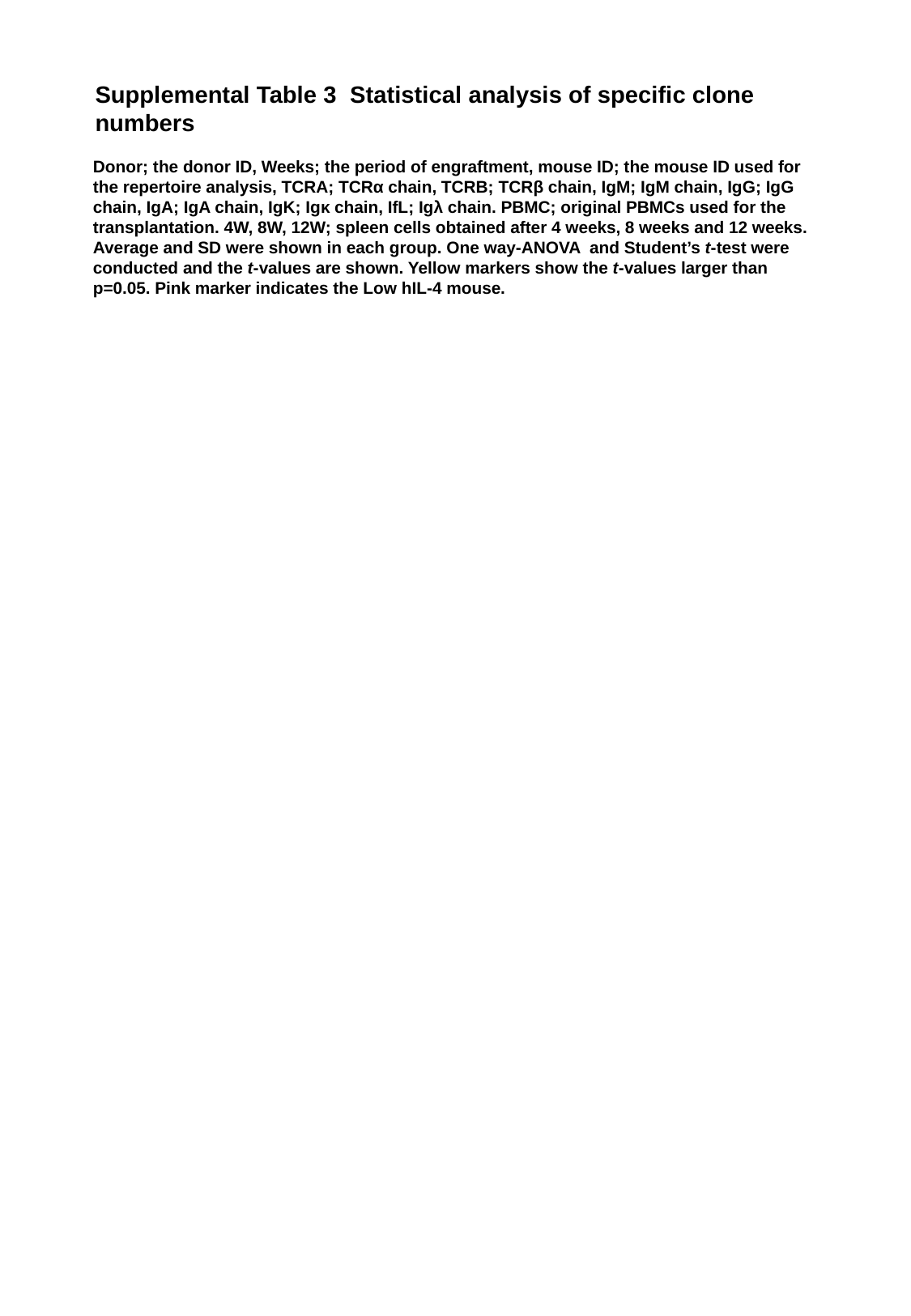

Supplemental Table 3 Statistical analysis of specific clone numbers
Donor; the donor ID, Weeks; the period of engraftment, mouse ID; the mouse ID used for the repertoire analysis, TCRA; TCRα chain, TCRB; TCRβ chain, IgM; IgM chain, IgG; IgG chain, IgA; IgA chain, IgK; Igκ chain, IfL; Igλ chain. PBMC; original PBMCs used for the transplantation. 4W, 8W, 12W; spleen cells obtained after 4 weeks, 8 weeks and 12 weeks. Average and SD were shown in each group. One way-ANOVA and Student’s t-test were conducted and the t-values are shown. Yellow markers show the t-values larger than p=0.05. Pink marker indicates the Low hIL-4 mouse.

## Slide 5
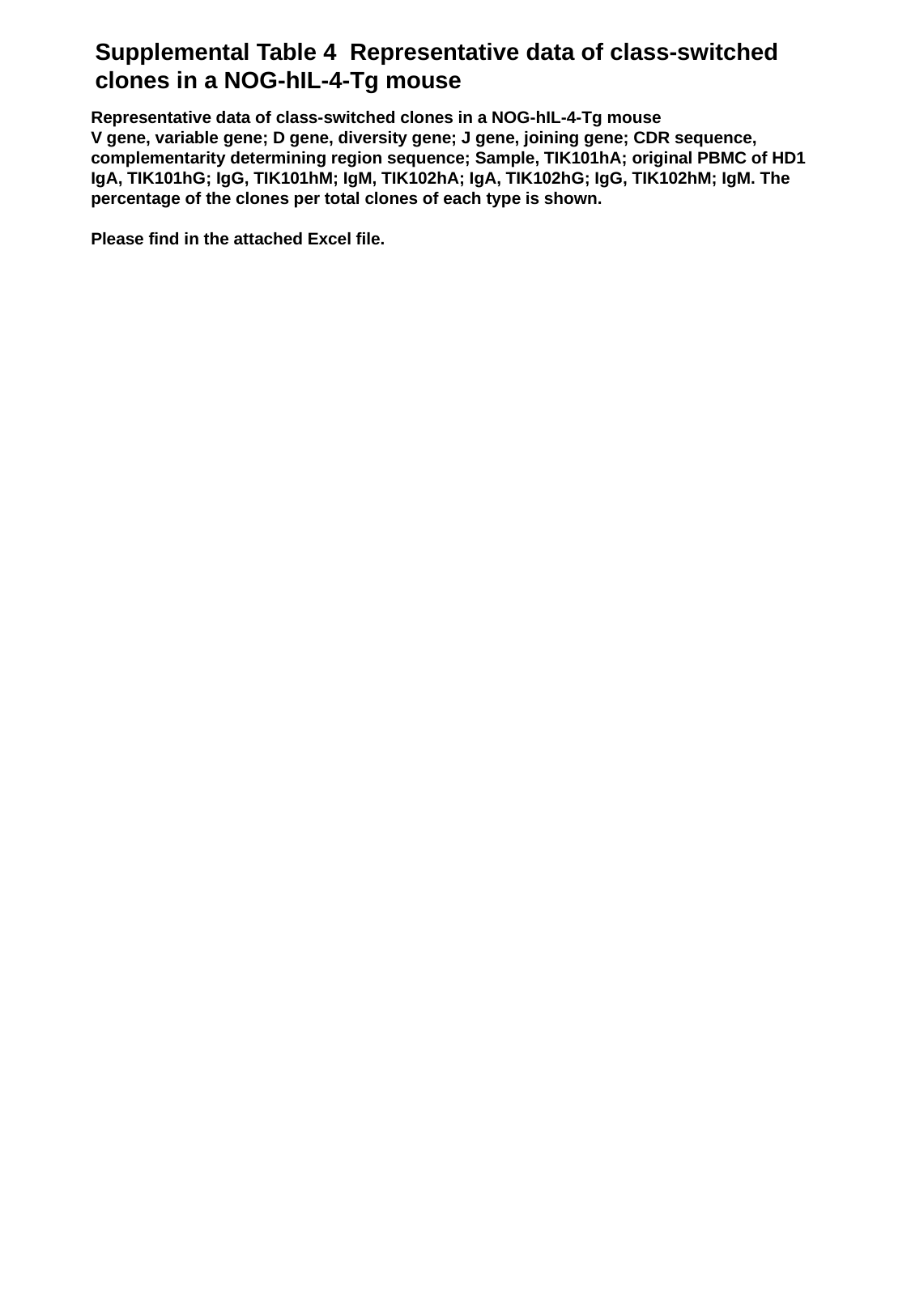

Supplemental Table 4 Representative data of class-switched clones in a NOG-hIL-4-Tg mouse
Representative data of class-switched clones in a NOG-hIL-4-Tg mouse
V gene, variable gene; D gene, diversity gene; J gene, joining gene; CDR sequence, complementarity determining region sequence; Sample, TIK101hA; original PBMC of HD1 IgA, TIK101hG; IgG, TIK101hM; IgM, TIK102hA; IgA, TIK102hG; IgG, TIK102hM; IgM. The percentage of the clones per total clones of each type is shown.
Please find in the attached Excel file.

## Slide 6
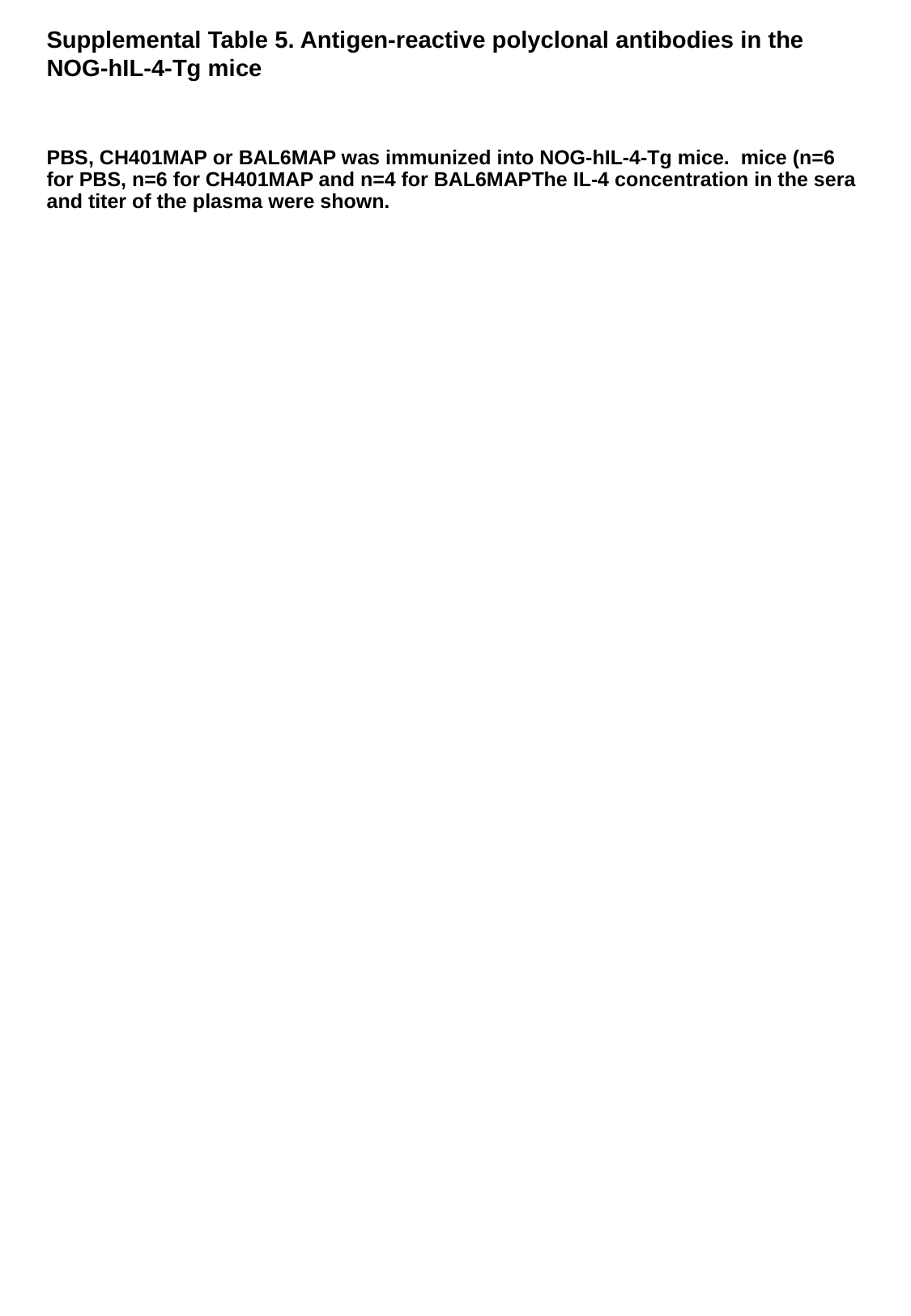

Supplemental Table 5. Antigen-reactive polyclonal antibodies in the NOG-hIL-4-Tg mice
PBS, CH401MAP or BAL6MAP was immunized into NOG-hIL-4-Tg mice. mice (n=6 for PBS, n=6 for CH401MAP and n=4 for BAL6MAPThe IL-4 concentration in the sera and titer of the plasma were shown.
